# Analyzing soccer match sprint distances: A comparison of GPS-based absolute and relative thresholds

**DOI:** 10.5114/biolsport.2024.133663

**Published:** 2024-02-07

**Authors:** Hugo Silva, Fábio Yuzo Nakamura, Irineu Loturco, João Ribeiro, Rui Marcelino

**Affiliations:** 1 Research Center in Sports Sciences, Health Sciences and Human Development, CIDESD, CreativeLab Research Community, Vila Real, Portugal; 2 University of Maia, Maia, Portugal; 3 Nucleus of High Performance in Sport, São Paulo, Brazil; 4 University of South Wales, Wales, United Kingdom; 5 Department of Performance Optimization, GOD, Sporting Clube de Braga SAD, Braga, Portugal; 6 Portugal Football School, Portuguese Football Federation, Oeiras, Portugal

**Keywords:** Competition, Football, Load, Speed, Team-sports, Velocity

## Abstract

This study compared the most common absolute sprint threshold (> 25.2 km/h) with relative and individualized thresholds (> 70%, > 75%, > 80%, > 85% and > 90% of peak match speed). Twenty elite soccer players, competing in the first division of the Portuguese League, were monitored using GNSS equipment during thirty-four official matches. Peak match speed was retrieved as the individual maximal speed reached during the full season. Distances were registered when speed overcame the absolute and the relative thresholds. Mean ± SD of peak speeds and distances covered were calculated, and Pearson correlation (*r*) and mean paired differences were performed to analyze relationships and differences between thresholds. The peak match speed was 32.9 ± 1.4 km/h. Correlations between distances covered using the absolute and relative thresholds varied from very strong (> 70%: *r* = 0.84, *p* < .001; > 75%: *r* = 0.89, *p* < .001; and > 80%: *r* = 0.88, *p* < .001), strong (> 85%: *r* = 0.79, *p* < .001), to moderate (> 90%: *r* = 0.59, *p* < .001). Overall, the > 75% (ES: 0.23 [95% CI: 0.16, 0.31]) and the > 90% (ES: -1.65 [95%CI: -1.85, -1.48]) relative thresholds presented the smallest and largest differences, respectively, with the absolute threshold. Differences were also found when considering the playing positions. While the distances covered by central midfielders were similar between the absolute and > 80% thresholds (-0.03 [-0.16, 0.10]), fullbacks covered largely more distance -1.88 [-2.42 -1.50]) in the absolute threshold than in the > 80% threshold. The distances covered by players varied based on the selected threshold, affecting the distances covered by different playing positions. Being the highest speed threshold within displacements thresholds, the absolute sprint threshold showed greater similarity to lower rather than higher relative thresholds.

## INTRODUCTION

With the advances in technological equipment (i.e., global positioning systems [GPS]) during the 1990’s, coaches and sport scientists began directing their attention to the analysis of athletes’ movements and efforts in training and competition [1]. Therefore, interesting data have been obtained regarding physical, physiological, and technical demands across technical-tactical training sessions and matches [2]. For example, it is known that soccer players usually cover between 9 and 14 km per match [3]. However, most of this distance (~35%) is covered at low speeds (< 7.2 km/h), with only 2–3% being covered at very-high speeds (> 25 km/h) [3]. Although practitioners continue to include total distance during match load monitoring [4], recent scientific research has focused on examining highintensity efforts such as sprint distance (distance covered above a specific threshold) [1]. This happens due to the characteristics of soccer, especially due to the high intermittency of match activities [2] that allows reaching multiple peaks of speed along the match. Sprint efforts has also gained a particular importance because these actions usually precede goal situations in official soccer matches [5]. On the other hand, these explosive actions are regularly associated with a high risk of injury, especially when players perform maximal sprints in a fatigued state [6, 7]. However, players constantly exposed to these efforts might be better prepared for specific match demands, thus reducing the injury risk [8].

When analyzing sprint efforts, researchers can provide interesting information regarding match demands. For instance, maximal sprint speeds (> 30 km/h) occur more often at the beginning and end of the match (i.e., 0’–15’ and 75’–90’; compared to the rest of the match) [9], with players covering between 30 and 55 meters at those speeds per match [10]. However, an important point should be raised when discussing this issue: a maximal effort is always related to the player’s aptitude and the context in which it occurs has critical implications. That is, maximal efforts might have distinct classifications and present a great variability among players of different fitness levels [11, 12]. In this regard, two interesting findings were recently reported. First, a study showed higher peak speeds in the 30-m sprint test compared to matches and sided games [13]. Secondly, arbitrary thresholds underestimated the distances covered when compared to relative thresholds (obtained using the 30–15 Intermittent Fitness Test, with peak speeds collected during training sessions and matches) [14]. Nevertheless, near-maximal efforts are usually classified according to arbitrary thresholds, without considering the individual characteristics of players and their respective playing positions [15]. For example, previous research has monitored sprint displacements with different absolute thresholds (i.e., distances covered > 19.8 km/h [8], > 21 km/h [16], > 24 km/h [17] and > 25.2 km/h) [18]. With these fixed ranges, two main issues arise: first, since speed thresholds differ between studies, comparisons between players’ efforts are difficult to be made; second, players with different physical and physiological capacities are evaluated under the same criteria. In practical terms, for example, whereas the 25 km/h threshold represents an intensity of ~71% for a player who reaches a maximum speed of 35 km/h, the same threshold represents an intensity of ~83% for another player who sprints at a maximum speed of 30 km/h.

To address these issues, some authors have proposed the use of a more individualized approach, with individual efforts being classified according to the maximum values obtained during field tests [16]. However, during matches, players rarely achieve speeds > 90% of individual maximum speed registered during tests [19]. Furthermore, field tests protocols may have limitations, such as underestimating peak speed when measuring average speed in 10 meters or 20 meters splits [20]. Hence, using peak match speed (i.e., the highest speed registered during the match) as the reference value for determining and classifying players’ efforts could provide a more realistic view of individual match demands. This strategy would benefit from analyzing the actual game context, accounting for players’ capacities and tasks, thereby overcoming the limitations of relying on speed values collected during sprint speed tests, which are usually higher to those attained during matches, as well as from using data collected during the pre-season period [13, 21]. Regarding the use of relative thresholds, a recent study revealed that the assessment of distances covered at speeds superior to 80% of match and training peak speeds allowed to differentiate between starters and non-starters [22]. Furthermore, distances covered at speeds > 25 km/h and > 80% of players’ maximum speeds differ, with players covering shorter distances while monitored with relative thresholds in comparison with absolute thresholds (i.e., starters and non-starters monitored with relative thresholds: ~940 m vs. ~550 m; starters and non-starters monitored with absolute thresholds: ~1300 m vs. ~970 m). Specifically, the 80% relative threshold has been recommended to ensure higher accuracy while monitoring sprinting activities [23]. However, the application of relative thresholds remains limited, as different relative intervals (> 80% and > 90%) are equally classified as sprints [20]. Therefore, the aim of this study was to analyze the relationships and differences between the distances covered during sprinting efforts of different intensities, classified according to the most commonly used absolute speed threshold for determining sprint efforts (> 25.2 km/h) [21] along with five relative and individualized speed thresholds (> 70%, > 75%, > 80%, > 85% and > 90% of peak match speed). Additionally, this study aims to compare the distances covered within each threshold based on the players’ positions. It was hypothesized that most of the individualized speed thresholds would result in significantly different sprinting distances compared to the absolute threshold, although the correlations among the distances covered would be significant. In addition, we also hypothesized that such differences (distances covered sprinting) would vary across playing positions.

## MATERIALS AND METHODS

### Study design

This retrospective study analyzed the relations and differences between distances covered during official matches of the Portuguese first division, according to specific thresholds, defined as absolute (> 25.2 km/h) (the most commonly used absolute threshold) and relative thresholds (> 70%, > 75%, > 80%, > 85% and > 90% of peak match speed), separated by 5% intervals. Data were collected across thirty-four matches (full season), from August 2021 to May 2022, following the standard procedures established by the soccer club. Data was retrieved as raw data, collecting speed (km/h) and distance (m) for each player and for each match.

### Subjects

Twenty elite-level soccer players [24] (age: 24.9 ± 4.0 years, height: 182.1 ± 7.5 cm, and body-mass: 75.1 ± 8.3 kg) competing in the first division of the Portuguese League were monitored during all matches of the League during the 2021/22 season. Matches from national cups and from the European competition were excluded. Data from goalkeepers and players who failed to complete at least one match were excluded. Only data for completed matches were considered, resulting in a total of 173 files. An a-priori power analysis was calculated using the G-Power software, which required 111 observations [25], to a respective power of 0.95. The number of completed matches per player ranged from 2 to 25. Importantly, since only full matches were included, the data were not influenced by potential noise from short (but intense) participation and performance of substitutes. Considering playing positions, were included: central defenders (CD) (*n* = 6), fullbacks (FB) (*n* = 3), central midfielders (CM)(*n* = 5), wide midfielders (WM) (*n* = 4), and forwards (FW) (*n* = 2). Ethics Committee clearance was obtained (35/2021) and the study was conducted in accordance with the Declaration of Helsinki.

### Procedures

Players were monitored with a 10-Hz global positioning system (Catapult Vector S7 – Catapult Sports, Melbourne, Australia) that encompassed a double constellation system (GNSS and GPS). This model has been certified by FIFA [26] and has demonstrated good reliability for peak speeds [27]. The horizontal dilution of precision (HDOP) for all observations was 1.02 ± 0.34, with an average of 12 ± 2 satellites acquired. Devices were positioned between the upper scapulae, at approximately the T3–4 junction, being activated 15 minutes before use, in accordance with the manufacturer’s instructions. Raw data regarding distance and speed (km/h) were retrieved from the proprietary software (OpenField Console, Catapult Sports, Melbourne, Australia).

Peak match speed was defined as the highest value attained by each player across the thirty-four matches. Individual relative thresholds were individually calculated as > 70%, > 75%, > 80%, > 85%, and > 90% of the individual peak match speed. Covered distances were counted for every 0.1 seconds if velocity surpassed the established thresholds (> 25.2 km/h, > 70%, > 75%, > 80%, > 85% and > 90% of peak match speed).

### Statistical Analysis

All analysis were performed using Microsoft Excel and Jamovi (Version 2.3.19.0; JAMOVI project, 2022) with the ESCI package [28, 29]. Means ± SD and coefficient of variation (%CV) of peak match speeds were calculated for each playing position and for all players. Means ± SD of distances covered by players according to the specific threshold (> 25.2 km/h and > 70%, > 75%, > 80%, > 85% and > 90% of peak match speed) were calculated for each playing position and for all players. Pearson (*r*) correlation (with 95% Confidence Intervals) analyzed the relationships between absolute and relative thresholds. Magnitudes of correlations were classified as very weak (0–0.19), weak (0.20–0.39), moderate (0.40–0.59), strong (0.60–0.79), and very strong (0.80–1) [30], and statistical significance was established at *p* < .05. Differences between measurement thresholds were analyzed via mean paired differences, with intra-individual comparisons for each threshold. For example, the distances covered within the absolute threshold by player A in match 1 were compared with the distances covered within the selected relative threshold by the same player in the same match. This analysis was performed with all players, divided according to playing positions. Effect sizes were established as trivial (< 0.2), small (0.2 < 0.6), moderate (0.6 < 1.2), large (1.2 < 2.0), very large (2.0 < 4.0), and huge (> 4.0), and presented along with 95% confidence intervals [31]. An unclear effect size was established if the CI crossed zero [32].

## RESULTS

**FIG. 1:**
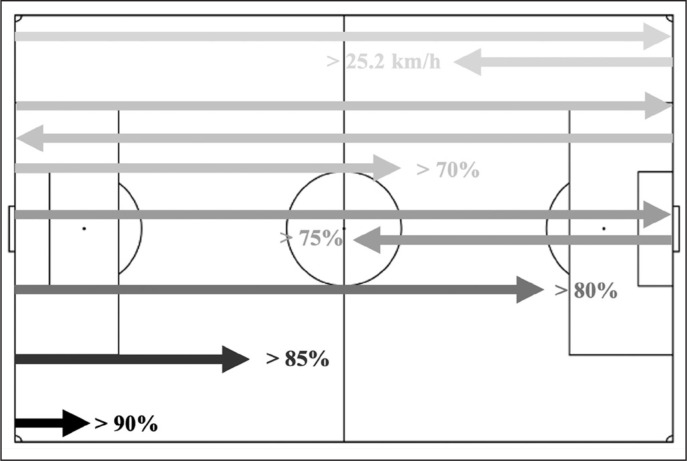
Distances covered per match according to the absolute threshold (> 25.2 km/h) and the relative thresholds (70%, > 75%, > 80%, > 85% and > 90% of match peak speed).

FW, FB, and WM reached higher peak match speeds across the full season and covered higher distances per match independent of the threshold (Table 1). Peak match speeds exhibited a %CV ranging from 0.9% (FW) to 4.9% (WM). Additionally, the intra-individual %CV of peak match speeds across the matches ranged from 0.9% to 7.7%, while the %CV of the team’s peak match speeds was 4.3%. Distances covered using the absolute threshold were longer than the > 80% threshold for CD, CM, and WM, and the 75% threshold for FB and FW (Table 1). Overall, distance covered decreased when considering the following thresholds: > 70%, > 75%, > 25.2 km/h, > 80%, > 85% and > 90% (Figure 1).

**TABLE 1:** Mean ± SD of peak match speed and distances covered per match according to the threshold, for each playing position and for all players.

	Peak Match Speed (km/h)	Distances covered (m)					

		> 25.2 km/h	> 70% peak match speed	> 75% peak match speed	> 80% peak match speed	> 85% peak match speed	> 90% peak match speed
CD	32.3 ± 1.0	92.7 ± 47.8	211.8 ± 68.2	121.9 ± 52.2	63.5 ± 36.0	29.9 ± 22.2	8.7 ± 10.5
FB	34.1 ± 0.7	258.0 ± 72.9	371.40 ± 77.1	231.9 ± 65.4	134.3 ± 52.4	63.5 ± 32.3	22.9 ± 19.8
CM	31.7 ± 0.7	44.7 ± 30.8	205.9 ± 83.1	102.1 ± 51.4	43.9 ± 28.0	15.4 ± 15.0	4.4 ± 7.1
WM	33.4 ± 1.6	185.8 ± 115.6	361.5 ± 109.2	212.7 ± 82.0	111.6 ± 54.0	49.6 ± 34.3	15.4 ± 19.8
FW	34.3 ± 0.3	244.7 ± 105.1	349.2 ± 119.8	204.6 ± 81.8	109.2 ± 44.1	46.0 ± 24.6	15.7 ± 11.7
All	32.9 ± 1.4	134.5 ± 103.5	270.3 ± 113.2	156.4 ± 80.8	82.0 ± 52.6	36.4 ± 30.1	11.7 ± 15.2

Note: CD = central defenders; FB = fullbacks; CM = central midfielders; WM = wide midfielders; FW = forwards

**FIG. 2:**
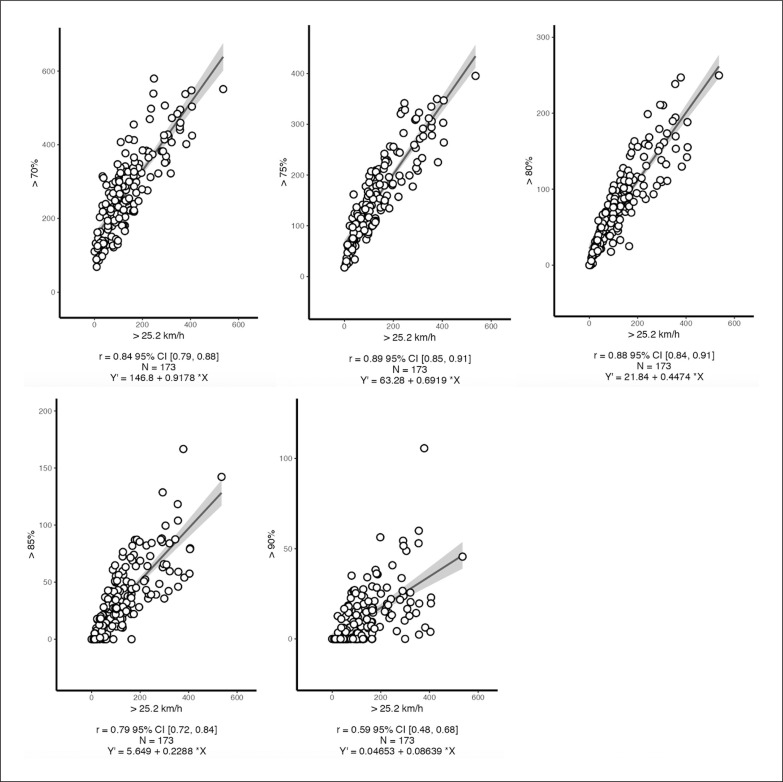
Pearson correlations (*r*) between the absolute threshold (> 25.2 km/h) and the relative thresholds (70%, > 75%, > 80%, > 85% and > 90% of peak match speed).

Very strong correlations were found between the absolute threshold and > 70% (*r* = 0.84, *p* < .001), > 75% (*r* = 0.89, *p* < .001), and > 80% (*r* = 0.88, *p* < .001) relative thresholds (Figure 2). Strong and moderate correlations were found between the absolute threshold and > 85% (*r* = 0.79, *p* < .001), and > 90% (*r* = 0.59, *p* < .001) relative thresholds, respectively (Figure 2).

Table 2 presents the mean differences according to the different thresholds and for each playing position and all players. Overall, the > 75% relative threshold presented the smallest difference (ES: 0.23 [90% CI: 0.16, 0.31]) when compared with the absolute threshold. Actually, on average, the 25.2 km/h threshold represented 76.8% of the peak match speed, ranging from 70.4% to 81.8%. However, for CM, the > 80% relative threshold presented unclear differences (ES: -0.03 [90% CI: -0.16, 0.10]) with the absolute threshold. The absolute threshold differed largely to hugely from the > 70% (except forwards which differed moderately) and the > 90% relative thresholds.

**TABLE 2:** Mean differences [90% CI] with effect sizes [90% CI] of distances covered per match between the absolute and relative sprint thresholds (> 70%, > 75%, > 80%, > 85% and > 90% of peak match speed) for each playing position and for all players.

	Distances covered (m)				

	> 70% peak match speed	> 75% peak match speed	> 80% peak match speed	> 85% peak match speed	> 90% peak match speed
CD	119.06 [107.69, 130.42]	29.19 [22.17, 36.22]	-29.22 [-34.88, -22.57]	-63.79 [-72.20, -55.38]	-84.04 [-94.07, -74.00]

	2.00 [1.73, 2.32]^VL^	0.58 [0.43, 0.74]^S^	-0.68 [-0.86, -0.53]^M^	-1.69 [-1.98, -1.45]^L^	-2.40 [-2.79, -2.08]^VL^

FB	113.36 [96.86, 129.86]	-26.09 [-42.41, -9.78]	-123.74 [-141.80, -105.68]	-194.58 [-218.57, -170.60]	-235.18 [-263.66, 206.71]

	1.46 [1.15, 1.89]^L^	-0.36 [-0.61, -0.15]^S^	-1.88 [-2.42, -1.50]^L^	-3.33 [-4.24, -2.71]^VL^	-4.25 [-5.42, -3.44]^H^

CM	161.19 [138.35, 184.04]	57.40 [47.51, 67.29]	-0.80 [-4.87, 3.28]	-29.30 [-37.94, -20.66]	-40.24 [-50.57, -29.92]

	2.50 [2.07, 3.10]^VL^	1.32 [1.08, 1.65]^L^	-0.03 [-0.16, 0.10]^U^	-1.18 [-1.57, -0.86]^M^	-1.75 [-2.29, -1.34]^L^

WM	175.71 [146.84, 204.58]	26.92 [3.13, 50.72]	-74.23 [-101.36, -47.09]	-136.14 [-169.13, -103.16]	-170.36 [208.26, -132.46]

	1.52 [1.20, 1.94]^L^	0.26 [0.06, 0.48]^S^	-0.80 [-1.07, -0.59]^M^	-1.55 [-1.97, -1.24]^L^	-2.00 [-2.54, -1.59]^VL^

FW	104.45 [78.12, 130.78]	-40.07 [-62.21, -17.93]	-135.48 [-184.71, -86.24]	-198.70 [-264.16, -133.25]	-228.97 [-306.43, -151.51]

	0.84 [0.59, 1.30]^M^	-0.39 [-0.62, -0.25]^S^	-1.53 [-2.30, -1.11]^L^	-2.36 [-3.60, -1.70]^VL^	-2.78 [-4.41, -1.84]^VL^

All	135.75 [126.38, 145.12]	21.82 [14.42, 29.23]	-52.51 [-61.91, -43.12]	-98.11 [-110.44, -85.78]	-122.87 [-137.22, -108.53]

	1.25 [1.13, 1.38]^L^	0.23 [0.16, 0.31]^S^	-0.64 [-0.73, -0.55]^M^	-1.28 [-1.43, -1.15]^L^	-1.65 [-1.85, -1.48]^L^

Note: CD = central defenders; FB = fullbacks; CM = central midfielders; WM = wide midfielders; FW = forwards; when the value is negative, it indicates that players covered greater distances at speeds exceeding the absolute sprint threshold (> 25.2 km/h); U = unclear effect size; S = small effect size; M = moderate effect size; L = large effect size; VL = very large effect size; H = huge effect size.

## DISCUSSION

The aim of this study was to compare distances covered by soccer players, per match, according to the most commonly used speed threshold for determining sprint efforts (> 25.2 km/h) with five other relative speed thresholds (> 70%, > 75%, > 80%, > 85% and > 90% of peak match speed). Our main finding agreed with our hypothesis, showing that sprint distances differ greatly according to the selected threshold, and with players covering more than one soccer field (i.e., 134.5 ± 103.5 m) per match at speeds > 25.2 km/h. This is longer than the distances covered at speeds > 80% of the individual peak match speed (i.e., +64%) but shorter than the distances covered at speeds > 75% (i.e., -14%) of the individual peak match speed (Figure 1).

Absolute speed thresholds allow comparisons within and between players and teams [33]. However, these thresholds are arbitrarily defined, with authors reproducing previous research and practitioners adopting the speed thresholds (i.e., speed zones) as determined by the proprietary GPS software [15]. Furthermore, although absolute thresholds consider that all displacements above a specific speed represent a similar level of effort for all players, the use of this method does not provide an individually tailored and precise characterization of match load [22, 34]. To address this issue, individualized speed thresholds have been proposed, by calculating the percentage of effort according to the maximum speed test [34] or using the peak running speed recorded during matches or training sessions [22]. Considering that during matches players fail to achieve the maximum speed obtained during tests [13], the latter method can reflect a more realistic and applied scenario. Our findings relate to and expand the findings reported by Gualtieri et al. [22], with players, in general, covering shorter distances at individualized “speed thresholds” (> 80% peak match speed) compared to the absolute sprint threshold (> 25.2 km/h) (Figure 1). In contrast, Abbot et al. [34] reported lower percentages of sprint distances covered by players with the individualized threshold (> 30% of the anaerobic speed reserve [the difference between the maximum sprint speed and maximum aerobic speed score]), except for players with superior performance during the “maximum aerobic speed test” (i.e., > 1 standard deviation from mean). However, the latter study defined relative thresholds using test results and not using data collected during official matches.

Another potential issue when using absolute speed thresholds to map and monitor external load relates to how and to what extent the specific match demands differ across different playing positions. For instance, previous research has reported that CD (186 ± 82 m) and CM (167 ± 87 m) cover shorter sprint distances compared to FB (265 ± 121 m), WM (314 ± 123 m) and FW (345 ± 29 m) when using fixed and standardized speed thresholds [35]. Despite the similarity with our results, the magnitude of these differences decreases substantially when individualized speed thresholds are considered. For example, considering our findings, FB covered 5.8 × more sprint distance than CM when considering the absolute threshold (> 25.2 km/h) but only 3.1 × more sprint distance than CM when considering the > 80% relative threshold (Table 1).

Selecting the adequate speed threshold is important to ensure that data is being correctly analyzed and interpreted, according to its purposes. For example, the 80% relative threshold provided the closest speed (0.1 km/h of difference) compared to the absolute threshold (> 24.9 km/h), but more importantly, this relative threshold was more associated with risk of injury in comparison to the absolute speed threshold in Australian football rules players [23].

Increasingly, we found very close correlations between the absolute speed threshold (> 25.2 km/h) and the > 70% (*r* = 0.84, *p* < .001), > 75% (*r* = 0.89, *p* < .001), and > 80% (*r* = 0.88, *p* < .001) relative thresholds (Figure 2). However, it does not mean that they can be used interchangeably. As previously discussed, the absolute threshold represented an intensity of 70.4% to 81.8% of the peak match speed, highlighting why the higher correlations were found within these intervals. Although distances covered above absolute and arbitrary thresholds correlated very strongly with those covered above relative thresholds, player individuality is not considered by the former. Hence, absolute and relative thresholds can be used for different purposes. As aforementioned, absolute thresholds might be used to compare teams and playing positions. On the other hand, the relative thresholds are able to track changes in peak speed during training/matches over the season and provide a more accurate sprint training/match load.

During high-speed displacements monitoring process, the sprint threshold (i.e., > 25.2 km/h) is the highest absolute threshold used [21]. Therefore, it is crucial to consider players capabilities while assessing intense efforts such as sprints. In order to establish the maximal speed to determine the relative thresholds, we considered the peak speed achieved during matches instead of using data collected through standard speed tests (e.g., a 30-m linear sprint speed test). Although match context influences player performance [36], which limits our findings, field tests also present their inherent limitations. As previously discussed, field tests do not necessarily reproduce actual match situations [13] and are primarily performed during preseasons (which may compromise the use of these data across the competitive season). In this regard, to account for peak match speed variation across the season [37], we used the highest value of peak match speed recorded throughout the entire season during the competitive matches.

However, since sprint efforts refer to the highest threshold when assessing speed displacements, choosing a lower relative intensity (such as > 75% of the peak match speed) can overestimate players’ loads. In contrast, choosing a high relative intensity (such as > 90% of the peak match speed) can result in the absence of sprints for some players, as the 90% intensity can even not occur during matches if one considers the maximum speed registered during field tests [19]. Considering that the > 80% has been previously recommended [22, 23], and can potentially increase the total displacement considered within the “actual” sprint threshold, this relative threshold may be used to improve the accuracy and efficacy of monitoring procedures, especially if the relation between this threshold and the risk of injury found Australian football rules [23] is replicated in future studies regarding soccer. Importantly, we only assessed one team and, to avoid potential noise in data, we only considered full matches. This strategy intended to avoid potential changes in data caused by short and intense appearances of substitutes. Additionally, it is worth noting that even when considering that peak match speed can vary across the season, field tests are typically conducted during the pre-season, which might increase this potential limitation.

## CONCLUSIONS

In conclusion, we found very strong correlations between distances covered above the absolute threshold and above three relative thresholds (> 70%, > 75% and > 80%). This is probably explained because the absolute threshold represented an intensity ranging from 70.4% to 81.8% of the peak match speed, but this does not mean that they can be used interchangeably. Sprint distances registered during matches differ according to the selected threshold, increasing the importance of what threshold to use while monitoring match sprint demands. Finally, different playing positions covered different distances, but the relative thresholds reduced the magnitude of those differences.

These findings have a direct impact on the load monitoring of soccer players. First, the absolute threshold neglects the players’ individual capacities, although it allows comparison within teams and players. For instance, practitioners may wish to determine whether a player can reach a specific speed or assess if that player covers longer/shorter distances at or above that particular speed. However, for meaningful and real comparisons between players, individualizing players’ capacities and demands could be a more effective strategy. Although faster players (assessed during field tests) achieved higher match speeds [38], a tactical strategy from the team or from the opposition can limit the expression of those sprint test speeds. For this reason, it makes sense recording peak speeds during matches since they better express the competitive demands. Considering the differences found between the distances covered according to the absolute or relative thresholds, the match load would vary according to the chosen strategy, which can impact how practitioners prepare the following training sessions. Finally, the relative threshold reduces the magnitude of the differences found between playing positions, highlighting the players’ individual capacities and matches’ context.
